# LncRNA MIR22HG promotes osteoarthritis progression via regulating miR-9-3p/ADAMTS5 pathway

**DOI:** 10.1080/21655979.2021.1945362

**Published:** 2021-06-30

**Authors:** Hui Long, Qin Li, Zhenping Xiao, Bo Yang

**Affiliations:** a Department of Pain and Rehabilitation, The Second Affiliated Hospital of University of South China, Hengyang, P. R. China; b Department of Orthopedics, Affiliated Nanhua Hospital of University of South China, Hengyang, P. R. China

**Keywords:** Osteoarthritis, mir22hg, miR-9-3p, adamts5

## Abstract

Dysregulation of long non-coding RNAs (lncRNAs) plays a fundamental role in the development and progression of osteoarthritis (OA), but the potential functions of lncRNAs in OA were not fully clarified. In the present work, we want to clarify the underlying functions and mechanisms of MIR22HG in OA. qRT-PCR was employed to detect the mRNA expression of MIR22HG, miR-9-3p, and ADAMTS5, while the protein expressions were measured using Western blot. The cell proliferation was examined through CCK8, while apoptosis was used in flow cytometry. Luciferase reporter assay and RNA immunoprecipitation (RIP) assays were undertaken to investigate the binding relationship among MIR22HG, ADAMTS5, and miR-9-3p. MIR22HG was significantly overexpressed in OA cartilages, OA chondrocytes and IL-1β-induced chondrocytes. Functionally, MIR22HG knockdown promoted cell proliferation, suppressed apoptosis, and contributed to downregulation of MMP13 and ADAMTS5 and upregulation of COL2A1 and ACAN in IL-1β-stimulated chondrocytes. Mechanistically, bioinformatic analysis indicated that MIR22HG may serve as a sponge for miR-9-3p and ADAMTS5 may be a potential targeted gene for miR-9-3p, which were subsequently verified through a dual-luciferase reporter assay. Moreover, rescue experiments showed that MIR22HG participated in the regulation of chondrocytes proliferation, apoptosis, and degradation of extracellular matrix via miR-9-3p/ADAMTS5 pathway. In conclusion, our findings illuminated that inhibition of MIR22HG ameliorated IL-1β-induced apoptosis and ECM degradation of human chondrocytes through miR-9-3p/ADAMTS5 pathway, which may provide a potentially promising target for OA treatment.

## Graphical Abstract

**Figure:**
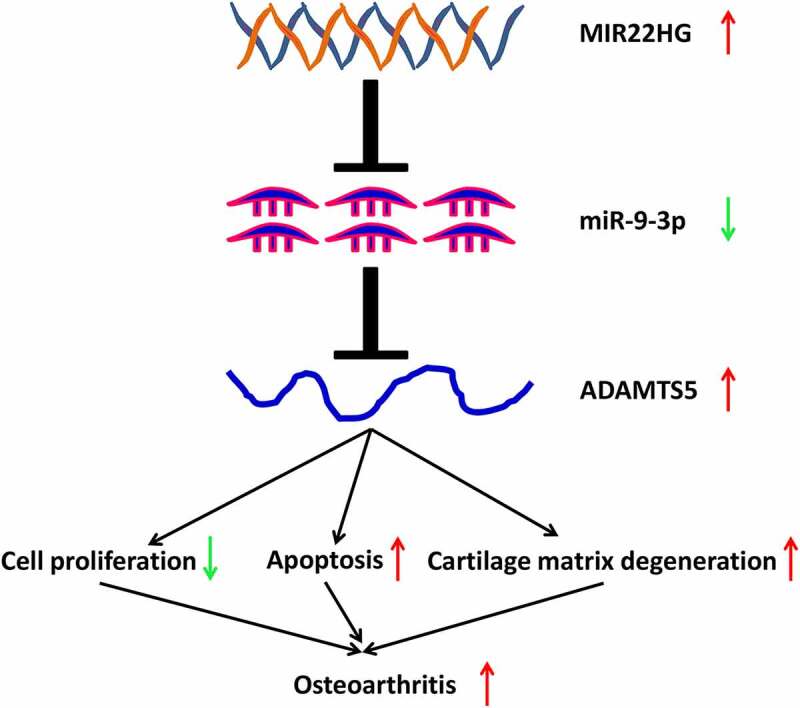

## Introduction

Osteoarthritis (OA), one of the most common joint degenerative diseases, is characteristic of progressive cartilage degradation, thickened subchondral bone as well as increasing bone spur formation [1]. With the aging of population and obesity epidemic, the prevalence of OA continues to rise up, which subsequently leads to serious social and medical burden [1,2]. Regardless of the advancements in OA treatment in recent these years, the current available medical therapy can hardly inhibit the progression of OA. Progressive extracellular matrix degeneration is the key pathological stage of OA [3,4], but the specific mechanisms for maintaining extracellular matrix homeostasis in OA are largely unclear. Therefore, it is necessary to further elucidate the potential mechanisms for extracellular matrix degeneration, which contributes to provide promising therapeutic targets in OA.

As a group of non-coding RNAs with nucleotides longer than 200, long non-coding RNAs (lncRNAs) were verified to be associated with the pathogenesis of several diseases [5,6]. Increasing studies showed that numerous lncRNAs were ectopically expressed in OA cartilage and participated in the onset and development of OA [7,8]. For instance, Wang and coworkers revealed that lncRNA XIST was markedly overexpressed in OA tissues. Moreover, XIST served as a sponge for miR-1277-5p to promote extracellular matrix degradation [9]. Gao and colleagues revealed that lncRNA MALAT-1 suppressed chondrocytes apoptosis and extracellular matrix degradation through JNK signaling pathway [10]. Irrespective of the emerging roles of many identified lncRNAs in OA progression, the clear functions of lncRNAs participating in OA pathogenesis were largely unknown. As a recently-indentified lncRNA, MIR22HG was found to be associated with the onset and progression of many cancers. Su and colleagues revealed that MIR22HG was apparently downregulated in lung cancer tissues as compared with paracancerous tissues. Furthermore, suppression of MIR22HG significantly accelerated lung cancer cell proliferation via YBX1, MET, and p21 in lung cancer [11]. Also, Zhang and colleagues revealed that MIR22HG suppressed malignant biological behaviors of hepatocellular carcinoma cells [12]. Han and coworkers demonstrated that MIR22HG was significantly upregulated in glioblastoma and it promoted the malignant phenotypes of glioblastoma through Wnt/β-catenin signaling pathway [13]. Regardless of the important roles of MIR22HG in cancer progression, studies involving the roles of MIR22HG in OA were still lacking. Interestingly, a previous RNA-seq data indicated that MIR22HG was significantly upregulated in damaged knee cartilage [14], but the possible roles and underlying mechanisms of MIR22HG in OA was elusive.

The current study aimed at clarifying the expression characteristics, potential roles, and mechanisms of MIR22HG in OA. We hypothesized that MIR22HG was highly expressed in the OA cartilage and MIR22HG may promote chondrocyte apoptosis and extracellular matrix degradation via miR-9-3p/ADAMTS5 pathway. Our work may reveal the important roles of MIR22HG in OA pathogenesis, which might provide a novel promising molecular target for OA treatment.

## Materials and Methods

### Articular cartilage specimens

Twenty human OA knee cartilage specimens were collected from the Affiliated Nanhua Hospital of University of South China between 2018.2 and 2021.2. All the patients met the clinical and radiological diagnostic criteria for OA. Articular cartilages were acquired from patients with OA undergoing total articular cartilage replacement, while 10 normal knee cartilage specimens from patients with trauma-induced amputation. All the patients in control group did not have OA or other arthritis history. Cartilage tissue specimens were immediately frozen in the liquid nitrogen. The current study obtained the Ethics Committee's approval of Affiliated Nanhua Hospital of the University of South China. All the participants signed written informed consents.

### Cell Culture

Randomly take six patients (OA or non-OA) to separate chondrocytes. Cartilage tissues were rinsed in phosphate buffered saline and cut into fragments (1 mm^3^). After digestion of trypsin (0.05%) for 20 minutes, centrifuged at 1000 r/min for 15 min, incubated with 0.1% type II collagenase and trypsin at 37°C for 4 h, primary chondrocytes were extracted from articular cartilage. Primary chondrocytes were cultivated in Dulbecco’s modified Eagle’s medium (DMEM; Gibco) containing 10% fetal bovine serum (FBS; Gibco), 100 IU/mL penicillin, and 100 mg/mL streptomycin in 5% CO_2_ incubator at 37°C. Only primary chondrocytes in the second or third generation were used in the current experiments, considering that chondrocytes may dedifferentiate after a few passages.

### Cell transfection

SiRNAs against MIR22HG (si-MIR22HG), miR-9-3p mimics, miR-9-3p inhibitors, ADAMTS5 overexpression plasmid, and their corresponding negative controls were synthesized and purchased from RiboBio (Guangzhou, China). Following the manufacturer’s protocol, these oligonucleotides were transfected into primary chondrocytes using Lipofectamine 2000 (Invitrogen, USA). The transfection efficiency was assessed 48 h after the transfection.

### qRT-PCR

TRIzol reagent (Invitrogen, USA) was employed to extract the total RNA in cartilage tissues and primary chondrocytes. Isolated RNAs were reverse-transcribed using TaKaRa (Otsu, Shiga, Japan). Furthermore, SYBR Green PCR Master Mix (Thermo Fisher Scientific, USA) was used to conduct quantitative real-time PCR (qRT-PCR) in the Biosystems 7300 Real-Time PCR system (ABI, USA). U6 and GAPDH were regarded as internal controls. The relative expressions of RNAs were estimated using the 2^−ΔΔCT^ method. The primer sequences of RNAs for qRT-PCR in the current study were listed in *Table S1*.

### Western Blot

We used RIPA buffer (Cell Signaling Technology, USA) to extract the total protein of primary chondrocytes. A total of 30 µg protein was loaded into SDS-PAGE gels and subsequently transferred into PVDF membranes (Beyotime). Those membranes were incubated using 5% skim milk (Beyotime) and primary antibodies including anti-COL2A1 antibody (Proteintech), anti-ACAN antibody (Abcam), anti-MMP13 antibody (Abcam), anti-ADAMTS5 antibody (Abcam), and anti-GAPDH antibody (Abcam). Furthermore, these membranes were incubated using secondary antibody (goat antirabbit IgG H&L (HRP) (Abcam) and goat antimouse IgG H&L (HRP) (ab205719, 1:2000, Abcam, UK)) for 1 hour at room temperature. An enhanced chemiluminescence substrate kit (Amersham Pharmacia Biotech, Little Chalfont, UK) was applied to visualize the protein in PVDF membranes.

### CCK-8

Chondrocytes proliferation was examined with CCK8 Kit (Dojindo). Primary chondrocytes were cultivated with 1 × 10^5^ cells per well on a 96-well plate. After transfection for 24, 48 and 72 h, a total of 10 μL CCK8 was added into chondrocytes and incubated for 2 h at 37°C. Subsequently, OD value was detected at 450 nm with Microplate Reader (Bio-Rad, USA).

### Flow cytometry

Chondrocytes apoptosis was measured using Annexin V-FITC/PI kit (BD Biosciences, USA). Primary chondrocytes were washed by cold PBS and subsequently resuspended using 1 × binding buffer. After adding PI and Annexin V-FITC, cell suspension was incubated at room temperature for 15 min. Furthermore, following the manufacturer’s instructions, chondrocytes apoptosis was measured using flow cytometry FACS Calibur instrument (BD Biosciences).

### Bioinformatics analysis

StarBase online database (starbase.sysu.edu.cn/) was used to predict the potential binding relationships between MIR22HG and miR-9-3p, while Targetscan (http://www.targetscan.org/vert_71/) for miR-9-3p and ADAMTS5.

### Luciferase Reporter Assay

The 3ʹUTR sequences of MIR22HG or ADAMTS5 containing wild-type or mutant-binding site of miR-9-3p were integrated into pmirGLO vector (Promega, USA) to form WT-MIR22HG or MUT-MIR22HG and WT-ADAMTS5 or MUT-ADAMTS5, respectively. Then, miR-9-3p or miR-NC were transfected into 293 T cells with Lipofectamine 2000 (Invitrogen, USA) in accordance with the manufacturer’s protocol. Furthermore, Luciferase Reporter Kit (Promega Corporation) was employed to examine the luciferase activity.

### RNA Immunoprecipitation(RIP) Assay

RIP was conducted with Imprint® RNA Immunoprecipitation Kit (Sigma-Aldrich) in accordance with the manufacturer’s protocol. RIP lysis was used to deal with harvested chondrocytes followed by incubating with magnetic beads containing Anti-Ago2 or Anti-IgG antibodies. Then, the enrichment levels of MIR22HG and miR-9-3p were examined using qRT-PCR.

### Statistical Analysis

The above experiments were performed in triplicate. Student’s t test was used for two comparisons. For more than two comparisons, one-way ANOVA was chosen. Continuous data are present using the mean ± SD. Correlation analysis was performed using Pearson analysis. Meanwhile, p value less than 0.05 was identified to be statistically significant. All statistical analyses in the current study were undertaken with Graphpad Prism 7.0.

## Results

### MIR22HG was significantly overexpressed in OA cartilage tissues and IL-1β-stimulated chondrocytes

**Figure 1.**
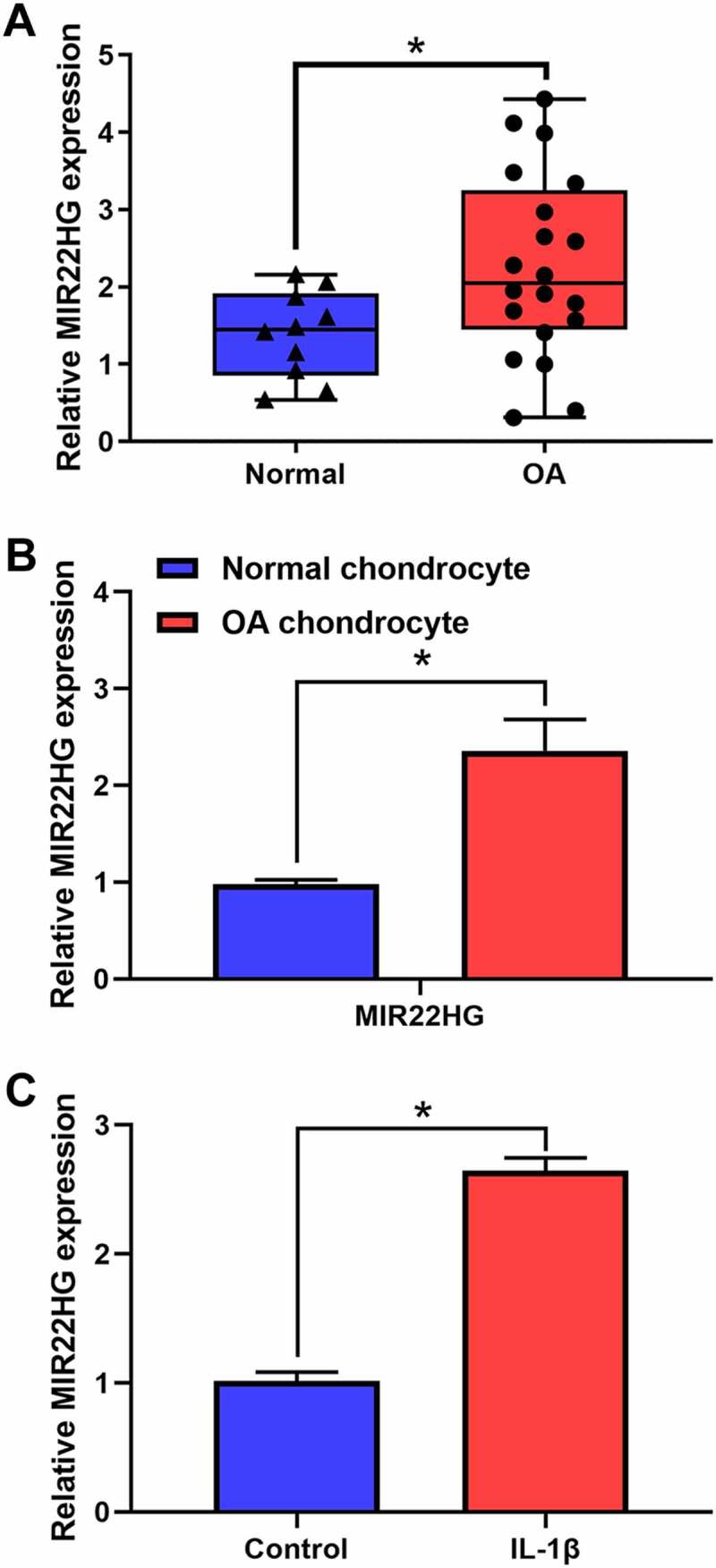
**MIR22HG was significantly overexpressed in OA**. (a) qRT-PCR was used to measure the expression level of MIR22HG in normal (n = 10) and OA cartilage (n = 20), *P < 0.05. (b) qRT-PCR was used to measure the expression level of MIR22HG in normal and OA chondrocyte. (c)The expression level of MIR22HG in IL-1β-stimulated chondrocyte detected using qRT-PCR. The ΔCt values were used to measure gene expression, which was normalized to the expression level of GAPDH. The results are shown as the mean ± SEM of at least three independent experiments (*P < 0.05)

To clarify the underlying functions of MIR22HG in OA, we firstly examined the expression level of MIR22HG in OA cartilage tissues and cell model. The results of qRT-PCR indicated that MIR22HG was obviously overexpressed in OA cartilage tissues and OA chondrocytes as compared with normal groups (Figure 1a, b). Furthermore, we used IL-1β (5 ng/mL) to treat primary chondrocytes. Similarly, the results of qRT-PCR also revealed that MIR22HG in IL-1β-stimulated chondrocytes was still higher than that in the non-treated group (Figure 1c). These findings revealed that MIR22HG was significantly upregulated in OA, which suggested that MIR22HG may be a potential contributor for OA.

**Suppression of MIR22HG inhibited IL-1β-induced apoptosis and extracellular matrix degradation of chondrocytes**

**Figure 2.**
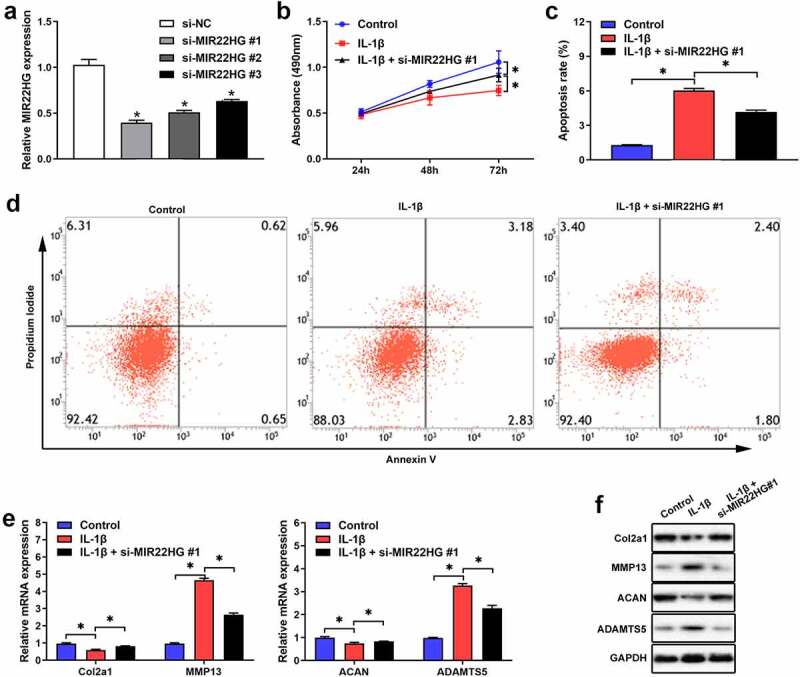
**Inhibition of MIR22HG accelerated cell proliferation and suppressed apoptosis and cartilage matrix degeneration in IL-1β-induced chondrocyte**. (a)The silence efficiency of si-MIR22HG were detected using qRT-PCR. CCK8, flow cytometry, qRT-PCR, and western blot were employed to measure chondrocyte proliferation(b), apoptotic (c, d), and the mRNA(e) and protein(f) expression level of COL2A1, ACAN, MMP13, ADAMTS5 in IL-1β-stimulated chondrocyte. The results are shown as the mean ± SEM of at least three independent experiments (*P < 0.05)

To further clarify the functions of MIR22HG in OA, we transfected si-MIR22HG into primary chondrocytes. qRT-PCR verified that si-MIR22HG#1 showed the higher silence efficiency (Figure 2a), so it was chosen for subsequent experiments. The results of CCK-8 and flow cytometry indicated that knockdown of MIR22HG significantly facilitated proliferation and repressed cell apoptosis in IL-1β-induced chondrocyte (Figure 2b-d). Also, qRT-PCR and Western blot further revealed that the expression levels of ADAMTS5 and MMP13 were significantly inhibited, while the expression levels of ACAN and COL2A1 were apparently upregulated after MIR22HG inhibition in IL-1β-induced chondrocyte (Figure 2e, f). These findings indicated that suppression of MIR22HG accelerated cell proliferation and repressed cell apoptosis and extracellular matrix degradation in IL-1β-induced chondrocyte.

### MIR22HG was a sponge of miR-9-3p in chondrocytes

**Figure 3.**
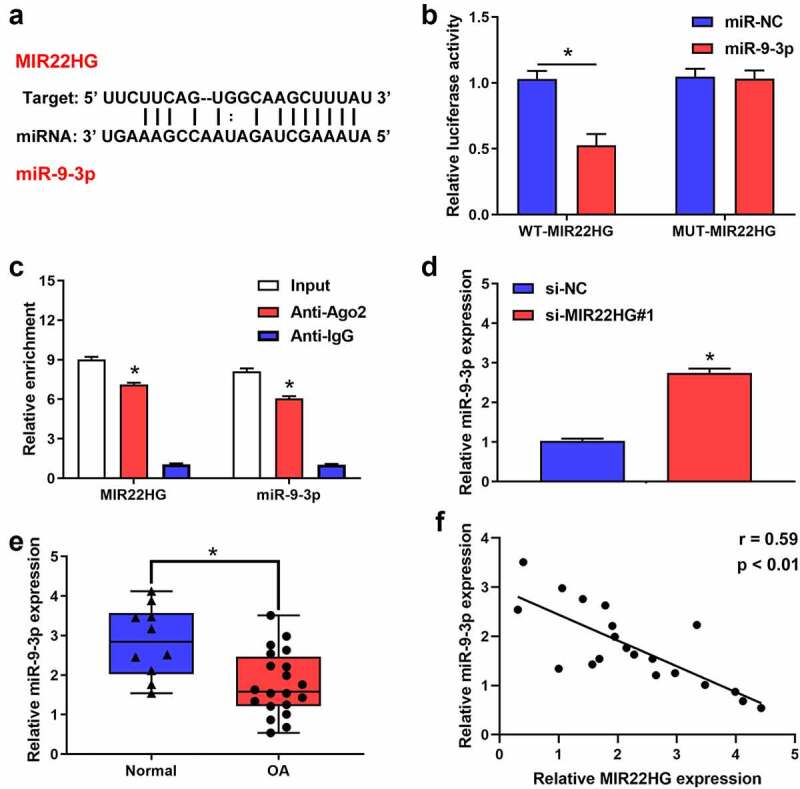
**MIR22HG targeted miR-9-3p in chondrocyte**. (a)The possible complementary sequences between MIR22HG and miR-9-3p was predicted by Starbase. (b)The luciferase activities in 293 T cells was examined using luciferase reporter assay after transfecting MIR22HG-wt and miR-9-3p mimic. (c) The relative enrichment of MIR22HG and miR-9-3p were examined using RIP experiments. (d)qRT-PCR was used to detect the expression level of miR-9-3p after silencing of MIR22HG. (e) The expression level of miR-9-3p in OA and normal cartilage were measured using qRT-PCR, *P < 0.05. (f)The correlation between the expression of MIR22HG and miR-9-3p in OA cartilage was estimated using Pearson analysis. The results are shown as the mean ± SEM of at least three independent experiments (*P < 0.05)

Increasing evidence indicated that MIR22HG could act as a sponge for several miRNAs, so we inferred that MIR22HG may work via interacting with miRNAs. To test this hypothesis, we performed bioinformatics analysis using Starbase to identify the potential miRNAs targeted by MIR22HG. Using the bioinformatics tool database, we predicted that there are several miRNAs that potentially bind to MIR22HG. Of these, we identified that miR-2467-5p, miR-4436a-3p, miR-5000-3p, hsa-miR-9-3p, miR-24-3p, miR-4756-5p, miR-1321, miR-370-3p and miR-6893-3p may be the most potential binding targets for MIR22HG after inhibited MIR22HG. Accordingly, RT-qPCR revealed that miR-9-3p exhibited the greatest change (Figure S1). Interestingly, previous studies revealed that miR-9-3p might serve as a possible protective factor in OA patients [15]. Also, we identified that MIR22HG contained the potential complementary points for miR-9-3p (Figure 3a). Accordingly, we wondered whether MIR22HG could act as a competitive endogenous RNA for miR-9-3p. Luciferase experiment found that miR-9-3p overexpression significantly reduced the relative luciferase activity of MIR22HG WT group, but no significant difference in MIR22HG MUT group (Figure 3b). Additionally, RIP assay indicated that MIR22HG and miR-9-3p significantly enriched in Ago2 pellet (Figure 3c). Meanwhile, qRT-PCR verified that si-MIR22HG#1 induced the upregulation of miR-9-3p (Figure 3d). Also, miR-9-3p was obviously downregulated in OA cartilage as compared with normal cartilage (Figure 3e). Furthermore, miR-9-3p was negatively correlated with the expression of MIR22HG (figure 3f). Taken together, these results suggested that miR-9-3p served as a downstream target of MIR22HG in OA.

**miR-9-3p inhibited IL-1β-induced apoptosis and extracellular matrix degradation** of chondrocytes **via ADAMTS5**

**Figure 4.**
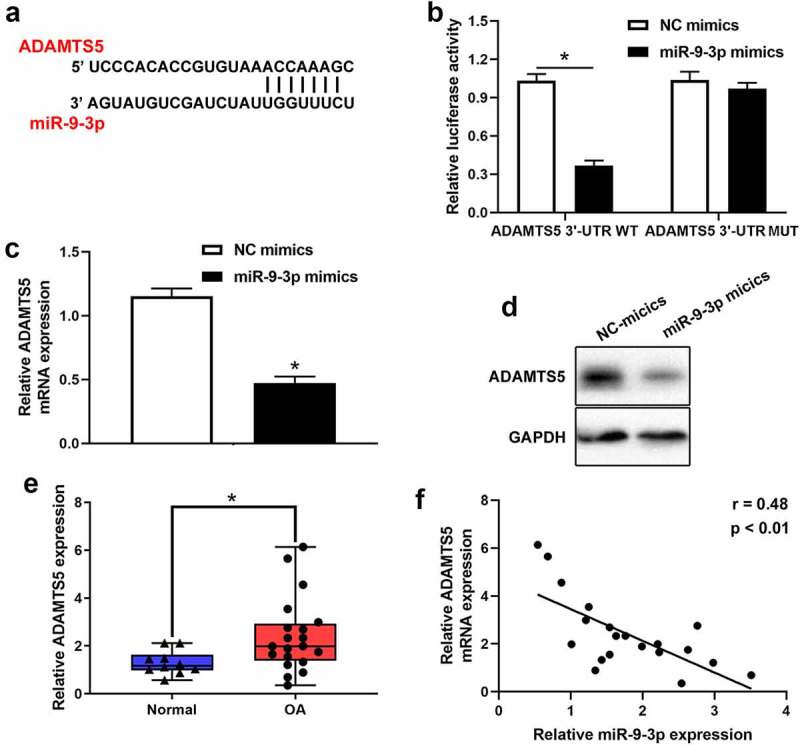
**ADAMTS5 acts as a downstream targeted mRNA of miR-9-3p**. (a)The possible binding sites between ADAMTS5 and miR-9-3p was predicted using Targetscan. (b)Luciferase reporter was used to validate the binding relationship of ADAMTS5 and miR-9-3p in 293 T cells. qRT-PCR(c) and western blot(d) were employed to measure the expression of ADAMTS5 after transfecting miR-9-3p mimics and NC. (e)The expression level of ADAMTS5 in OA and normal cartilage was examined using qRT-PCR. (f) The correlation between miR-9-3p and ADAMTS5 in OA cartilage was estimated using Pearson analysis. The results are shown as the mean ± SEM of at least three independent experiments (*P < 0.05)

**Figure 5.**
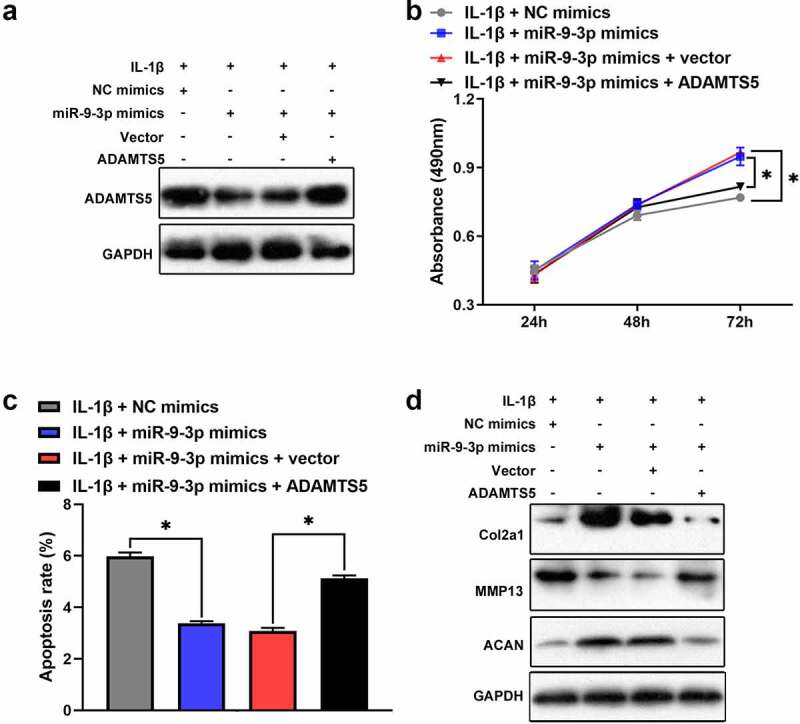
**miR-9-3p targets ADAMTS5 to inhibit IL-1β-triggered chondrocytes injury**. CCK8, flow cytometry, and western blot were employed to measure the protein expression of ADAMTS5(a), chondrocytes proliferation(b), apoptosis(c), and the expression level of COL2A1, ACAN, ADAMST5 and MMP13 (d) in IL-1β-induced chondrocyte following transfected with miR-9-3p mimics or ADAMTS5. The results are shown as the mean ± SEM of at least three independent experiments (*P < 0.05)

Based on Targetscan prediction, we identified that ADAMTS5 existed possible binding sites of miR-9-3p (Figure 4a). The result of luciferase reporter assay verified that miR-9-3p overexpression reduced the relative luciferase activity in ADAMTS5 WT group, but no significant difference in ADAMTS5 MUT group (Figure 4b). Furthermore, the results of qRT-PCR and western blot illustrated that the mRNA and protein level of ADAMTS5 was strikingly attenuated after miR-9-3p overexpression (Figure 4c, d). Also, qRT-PCR showed that ADAMTS5 was significantly upregulated in OA cartilage (Figure 4e). Meanwhile, ADAMTS5 was negatively correlated with the expression level of miR-9-3p (figure 4f). Collectively, these results indicated that ADAMTS5 acted as a downstream target of miR-9-3p. Furthermore, we identified that overexpression of miR-9-3p significantly surpressed the expression of ADAMTS5, which was partly reversed by ADAMTS5 overexpression (Figure 5a). Similarly, overexpression of miR-9-3p accelerated cell proliferation, which was partly restored by introduction of ADAMTS5 overexpression in IL-1β-treated chondrocytes (Figure 5b). Overexpression of miR-9-3p significantly antagonized cell apoptosis and degradation of extracellular matrix in IL-1β-stimulated chondrocytes, and these events were partly mitigated by restoration of ADAMTS5 (Figure 5c, d). Taken together, these results indicated that **miR-9-3p inhibited IL-1β-induced apoptosis and extracellular matrix degradation** of chondrocytes **via ADAMTS5**.

**Inhibition of MIR22HG attenuated IL-1β-induced apoptosis and extracellular matrix degradation** of chondrocytes **via miR-9-3p/ADAMTS5 axis**

**Figure 6.**
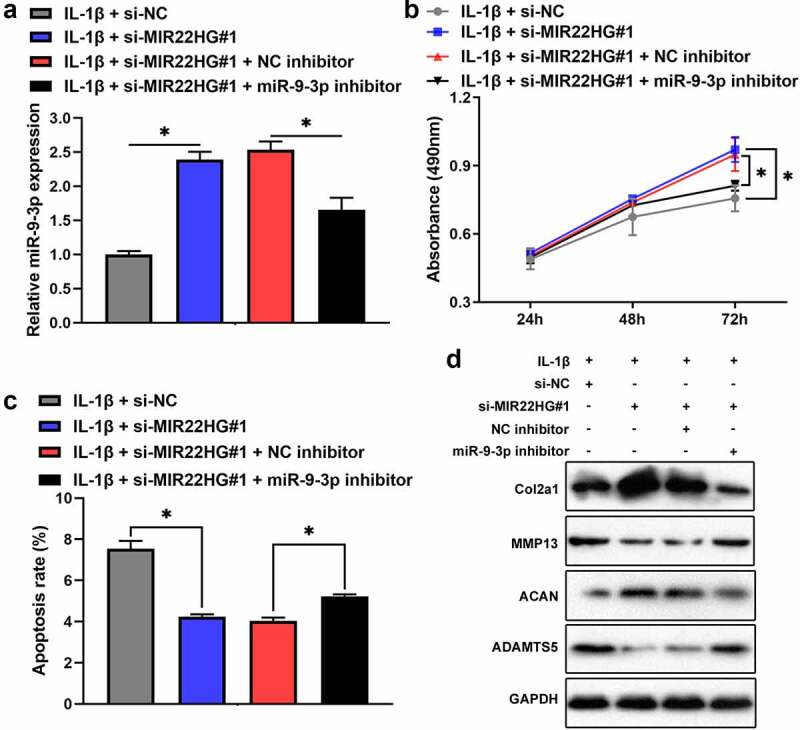
**Inhibition of miR-9-3p partly arrested the biological functions of MIR22HG knockdown on IL-1β-stimulated chondrocytes**. The mRNA level of miR-9-3p (a), cell proliferation(b), apoptosis(c), and the protein level of COL2A1, ACAN, MMP13, ADAMTS5 (d) were examined in IL-1β-stimulated chondrocyte after transfected with miR-9-3p inhibitor or si-MIR22HG using qRT-PCR, CCK8, flow cytometry, and western blot. The results are shown as the mean ± SEM of at least three independent experiments (*P < 0.05)

To elucidate mutual relationship among MIR22HG, miR-9-3p, and ADAMTS5, we transfected si-NC, si-MIR22HG#1, and si-MIR22HG#1 + miR-9-3p inhibitor into IL-1β-treated chondrocytes. qRT-PCR revealed that inhibition of MIR22HG promoted the expression of miR-9-3p, which was partly reversed by miR-9-3p inhibitor (Figure 6a). Consistently, depletion of MIR22HG apparently promoted chondrocyte proliferation and decreased chondrocyte apoptosis and degeneration of extracellular matrix in IL-1β-stimulated chondrocytes, which was partly recovered after transfecting miR-9-3p inhibitor (Figure 6b-d). Taken together, these results showed that **inhibition of MIR22HG attenuated IL-1β-induced apoptosis and extracellular matrix degradation** of chondrocytes **via miR-9-3p/ADAMTS5 axis**.

## Discussion

In the present work, our findings demonstrated that MIR22HG was significantly overexpressed in OA articular cartilages and IL-1β-stimulated chondrocytes. **Suppression of MIR22HG inhibited IL-1β-induced apoptosis and extracellular matrix degradation** of chondrocytes. Furthermore, we identified that **inhibition of MIR22HG attenuated IL-1β-induced apoptosis and extracellular matrix degradation** of chondrocytes **via miR-9-3p/ADAMTS5 pathway.**

Increasing studies showed that lncRNAs were associated with the onset and progression of many pathophysiological processes in several human diseases [16,17]. Also, previous studies demonstrated that MIR22HG participated in the onset and progression of many diseases. For instance, Jin and colleagues investigated that MIR22HG accelerated osteogenic differentiation of BMSCs via PTEN/AKT pathway [18]. Xu and coworkers revealed that MIR22HG inhibited the progression of colorectal cancer via TGFβ/SMAD signaling [19]. Regardless of the important roles of MIR22HG, the possible roles and molecular mechanisms of MIR22HG in OA was still elusive. In the current study, our findings verified that MIR22HG was significantly overexpressed in OA. Inhibition of MIR22HG accelerated chondrocyte proliferation, suppressed apoptosis and cartilage matrix degradation in OA chondrocytes. These findings demonstrated that MIR22HG could act as a significant contributor in the pathogenesis of OA.

Increasing studies demonstrated that MIR22HG exerted its regulatory functions by sponging miRNAs. Chen and colleagues found that MIR22HG inhibited malignant biological behaviors through miR-24-3p/p27kip1 pathway in thyroid papillary carcinoma [20]. Cui *et al*. announced that MIR22HG inhibited endometrial carcinoma cells proliferation via miR-141-3p/DAPK1 axis [21]. In our current work, our findings revealed that miR-9-3p served as the downstream target of MIR22HG. Actually, some studies verified that miR-9 had low expression in OA and acted as a potential protective factor in OA. Lee and colleagues revealed that miR-9 inhibited arthritis via NF-κB1-RANKL pathway in fibroblast-like synoviocytes [22]. Chen *et al*. also identified that upregulation of miR-9 suppressed chondrocytes apoptosis via Tnc in mice with OA [23]. Consistent with previous results, we also found that miR-9-3p was significantly downregulated in OA tissues and OA chondrocytes. Additionally, our findings verified that miR-9-3p inhibition could partly reverse the effects of MIR22HG silence on OA chondrocytes, which suggested that the protection effect for IL-1β-treated chondrocytes from MIR22HG inhibition may at least partly be achieved via miR-9-3p.

Generally, miRNAs exert their functions through binding targeted downstream genes. ADAMTS-5, as a important member of ADAMTS family, was found to participated in the initiation and progression of OA [24]. ADAMTS-5 is the major enzyme responsible for cartilage degradation in OA, so it may act as an promising therapeutic target for OA treatment. Actually, many studies revealed that some targets focused on ADAMTS5 could significantly ameliorate OA progression. Chu *et al*. found that intra-articular injection of ADAMTS-5 siRNA lentivirus inhibited the articular cartilage degradation in a rat OA model [25]. Also, Chen and coworkers demonstrated that the intra-articular injection of ADAMTS-5 inhibitor (114,810) ameliorated cartilage degeneration [26]. Our results demonstrated that ADAMTS5 was the downstream target for miR-9-3p. Furthermore, our findings indicated that downregulation of ADAMTS-5 was induced by MIR22HG inhibition through upregulating miR-9-3p, which suggested that MIR22HG exerted its regulatory function in OA via miR-9-3p/ADAMTS5 axis.

## Conclusions

Our findings demonstrated that MIR22HG was overexpressed in OA articular cartilage and may act as a significant contributor for OA progression. Moreover, MIR22HG participated in regulating the proliferation, apoptosis, and cartilage matrix degeneration via miR-9-3p/ADAMTS5 pathway. Therefore, these results suggested that MIR22HG/miR-9-3p/ADAMTS5 axis participated in the pathological progress of OA, which may provide a promising molecular target and biomarker for OA. In the following work, we will further collect OA clinical specimens to explore the relationship between MIR22HG and OA pathological stages or disease prognosis. Furthermore, animal models are established to explore the translational potentials of MIR22HG/miR-9-3p/ADAMTS5 axis in clinical treatment for OA.

## Supplementary Material

Supplemental Material

## Highlight

**1. MIR22HG was significantly overexpressed in OA cartilage tissues and IL-1β-stimulated chondrocytes.**

**2. Suppression of MIR22HG inhibited IL-1β-induced apoptosis and extracellular matrix degradation** of chondrocytes.

**3. Inhibition of MIR22HG attenuated IL-1β-induced apoptosis and extracellular matrix degradation** of chondrocytes **via miR-9-3p/ADAMTS5 axis**
