# Supplementary material for: LncRNA MIR22HG promotes osteoarthritis progression via regulating miR-9-3p/ADAMTS5 pathway
Source: Bioengineered. 2021 Jun 30;12(1):3148–58. doi: 10.1080/21655979.2021.1945362 (PMC8806551; doi:10.1080/21655979.2021.1945362)
Supplement: Supplemental Material [file KBIE_A_1945362_SM7053.zip › supplementary/Table S1.docx]

**Table S1. Primer sequences used in this study**

| Genes | Primer sequence | |
| --- | --- | --- |
| MIR22HG | Forward | AAGTTGGAGAGCCTTTGCCC |
|  | Reverse | CGCACTATGGTGCCACATCT |
| COL2A1 | Forward | TGGACGATCAGGCGAAACC |
|  | Reverse | GCTGCGGATGCTCTCAATCT |
| ACAN | Forward | ACTCTGGGTTTTCGTGACTCT |
|  | Reverse | ACTCTGGGTTTTCGTGACTCT |
| MMP13 | Forward | ACTGAGAGGCTCCGAGAAATG |
|  | Reverse | GAACCCCGCATCTTGGCTT |
| ADAMTS5 | Forward | GAACATCGACCAACTCTACTCCG |
|  | Reverse | CAATGCCCACCGAACCATCT |
| GAPDH | Forward | GGAGCGAGATCCCTCCAAAAT |
|  | Reverse | GGCTGTTGTCATACTTCTCATGG |
| miR-9-3p | Forward | GCGGCGGATAAAGCTAGATAAC |
|  | Reverse | ATCCAGTGCAGGGTCCGAGG |
| U6 | Forward | GCTTCGGCAGCACATATACTAAAAT |
|  | Reverse | CGCTTCACGAATTTGCGTGTCAT |
