# Supplementary figures and images for: LncRNA MIR22HG promotes osteoarthritis progression via regulating miR-9-3p/ADAMTS5 pathway
Source: Bioengineered. 2021 Jun 30;12(1):3148–58. doi: 10.1080/21655979.2021.1945362 (PMC8806551; doi:10.1080/21655979.2021.1945362)

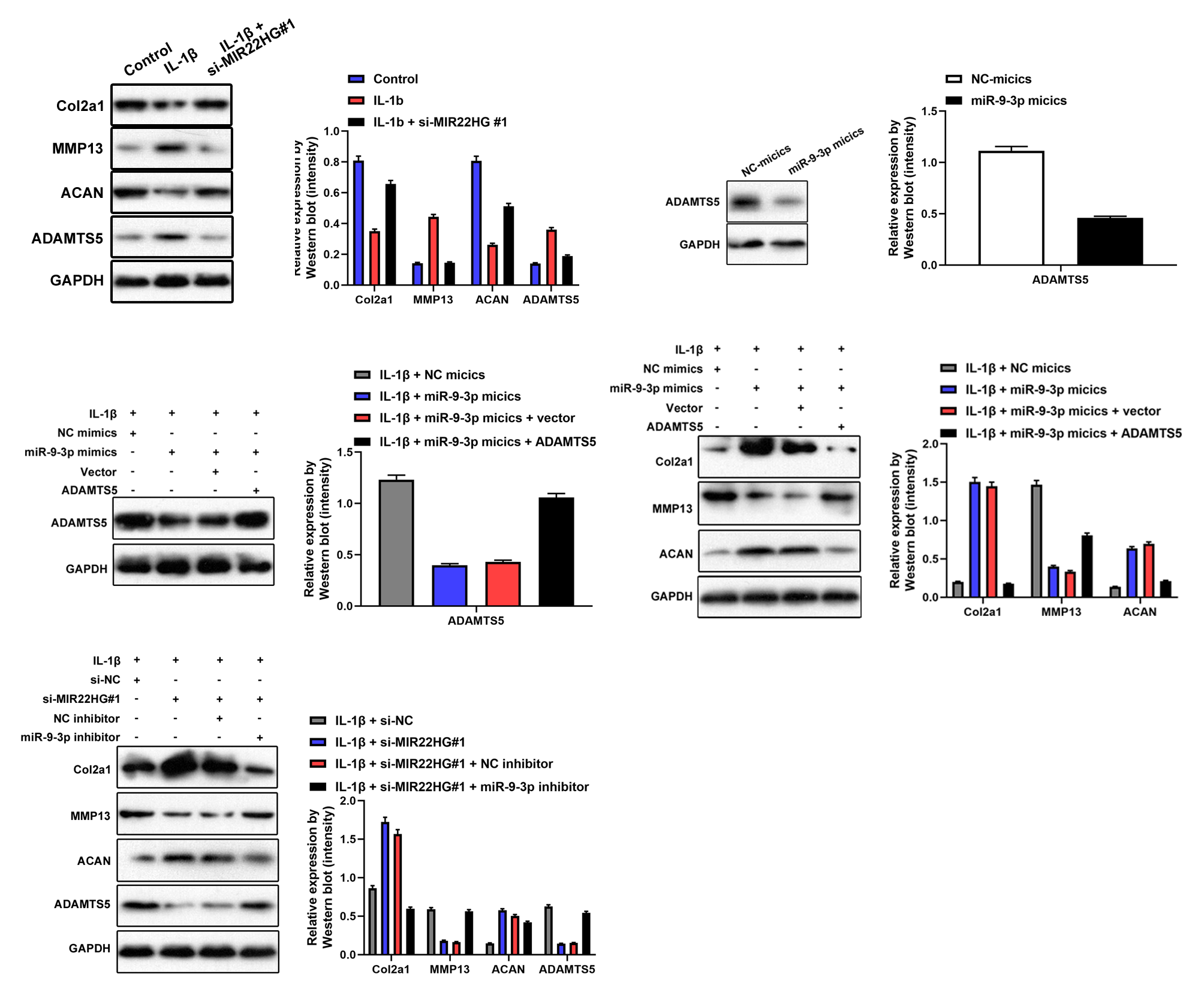

Supplement: Supplemental Material [file KBIE_A_1945362_SM7053.zip › supplementary/Figure S1.tif]
